# Cleaner technologies for asphalt mixtures combining reuse of residual aggregates, waste crumb rubber and warm mix asphalt additive

**DOI:** 10.1038/s41598-023-35235-z

**Published:** 2023-05-19

**Authors:** Miguel A. Franesqui, Ana María Rodríguez-Alloza, Jorge Yepes, Cándida García-González

**Affiliations:** 1 Grupo de Fabricación Integrada y Avanzada-Departamento de Ingeniería Civil, Universidad de Las Palmas de Gran Canaria (ULPGC), Las Palmas de Gran Canaria, Spain; 2 Grupo de Tecnología de Materiales en la Arquitectura y la Construcción (TermaCon)-Departamento de Ingeniería Civil, Náutica y Marítima, Universidad de La Laguna (ULL), San Cristóbal de La Laguna, Spain; 3 Departamento de Ingeniería Civil-IOCAG, Universidad de Las Palmas de Gran Canaria (ULPGC), Las Palmas de Gran Canaria, Spain

**Keywords:** Engineering, Civil engineering

## Abstract

The reuse of waste materials and residual aggregates as well as the reduction of emissions has become vitally important for the environment, the economy and logistics of the asphalt paving industry. This study characterizes the performance and production properties of asphalt mixtures with waste crumb-rubber modifier from scrap tires, a warm mix asphalt surfactant additive and residual poor-quality volcanic aggregates as the single mineral component. The combination of these three cleaner technologies provides a promising solution to produce more sustainable materials by reusing two different types of waste and decreasing the manufacturing temperature at the same time. The compactability, stiffness modulus and fatigue performance characteristics were assessed in the laboratory for different low production temperatures and compared to conventional mixtures. The results indicate that these rubberized warm asphalt mixtures with residual vesicular and scoriaceous aggregates comply with the technical specifications for paving materials. The dynamic properties are maintained or even improved while reusing waste materials and allowing reductions of the manufacturing and compaction temperatures up to 20 °C, therefore, decreasing energy consumption and emissions.

## Introduction

Aggregates constitute one of the most consumed resources in the world (more than 48 billion tonnes/year) and are fundamental construction materials. In fact, they are the main component (more than 90% by weight and more than 80% of the volume) in the manufacturing of asphalt mixtures for paving. Sometimes, high quality aggregates need to be transported over long distances, resulting in time loss and expenses as well as an environmental impact. For this reason, regional resources and waste aggregates should preferably be used for construction. This issue appears to be of special significance in volcanic areas as it is difficult to finding certain raw materials: the most abundant lithotypes are high-porosity vesicular and scoriaceous rocks^1,2^. These types of aggregates have different drawbacks that could make their use unfeasible. First, there is an excessive variability and heterogeneity due to the discontinuous and irregular distribution of lava flows and pyroclastic deposits. Secondly, these aggregates show high levels of absorption and low percentage of broken and crushed surfaces as they present a greatly porous alveolar structure^1^. A higher consumption of binders (or cement) and energy to evaporate water, and increased weathering, are consequences of the extreme porosity of these volcanic aggregates when manufacturing asphalt or cement concrete. This leads to inferior performance of these construction materials compared to conventional aggregates. Due to these aforementioned reasons, an important percentage of the aggregate production from quarries and deposits in volcanic areas is discarded, generating serious environmental problems. However, as aggregates are a depleting resource, the use of lower-quality or marginal aggregate sources is necessary even when they do not meet the technical specifications for road materials^3^. Hence, the reuse of local waste aggregates is becoming a requirement for the environment, the economy and logistics and certain techniques should be investigated in order to improve their properties.

Despite the necessity of using these marginal aggregates in volcanic regions, there are not many studies regarding their application in asphalt concrete mixtures and when studied, these investigations only refer to specific lithotypes such as volcanic ashes and trachybasalts^4–6^. Even though the most abundant aggregates in volcanic regions are vesicular and scoriaceous ones, very few publications have investigated the performance of bituminous mixtures that incorporate these aggregates. Other previous studies have shown that volcanic aggregates like basalt stone can be incorporated in asphalt mixtures^7^ and some researchers have compared basalt with calcareous aggregates or have studied the combination of basalt and limestone^8,9^.

On the other hand, the development of energy-efficient and environmentally friendly pavement technologies is pivot in the pavement engineering industry. To produce more sustainable materials for road construction, greenhouse gases emissions must be reduced as well as the use of resources by using waste materials. Warm mix asphalt (WMA) has been proposed and implemented in asphalt paving as a technology that allows to produce and compact asphalt mixtures at reduced temperatures in comparison to hot mix asphalt (HMA) without compromising the workability and the mechanical performance of the mixtures. As energy consumption is reduced in the asphalt mixing plant, emissions are decreased as well. Even more, workability is enhanced, binder aging is reduced, and construction time and hauling distances during works can be extended^10–12^. WMA can be classified into three different categories: foaming technologies, organic additives, and chemical agents. While foaming consists in including a certain amount of pulverized water either added directly to the binder or injected into the mixing tank^13^, organic additives act by reducing the viscosity of the base asphalt binder due to the presence of waxes^14,15^. Chemical agents include surfactants, emulsifiers, polymers or a combination of additives. These additives enhance the mixture workability and compaction, and improve binder coating over the aggregates^16,17^.

Rubberized asphalt is another sustainable technology that can contribute to solving the well-known environmental problem of waste used tires, especially in environmentally protected or limited territories. It has been shown that bituminous mixtures made with crumb rubber from end-of-life tires (ELT) offer excellent performance since, among other benefits, they increase resistance to permanent deformation and fatigue^18–21^. When the recycled rubber from ELT is added to the neat bitumen, the viscosity of the blend becomes much higher than the conventional binder^22,23^ and thus, increases the temperature required for the production and compaction of the asphalt mixtures. There is a high-temperature interaction of the bitumen with the rubber particles from the tire entails. A chemical reaction takes place, which consists in the degradation of the elastomeric chains due to thermal effect as well as the devulcanization of the rubber^24^. During this process, the destruction of sulphur bonds typical of vulcanization is evidenced by a decrease in sulphur on the surface of the rubber particles^25^. This rubber-bitumen interaction is called digestion and, during this process, the rubber particles begin to swell or increase in volume due to the absorption of the aromatic fractions of the bitumen^26^. The correct combination of digestion time and temperature ensures the integration of the rubber in the bitumen, improving its properties, although if the interaction is prolonged excessively, the improvement achieved is reduced by the dilution of the rubber in the bitumen. Lately, research efforts have focused on increasing the performance of this technique by reducing the production temperature of rubberized mixtures. This is the reason why coupling rubberized asphalt with WMA technology will be of great significance for the sustainability of these paving materials^27^.

Previous works shown that the effect of rubberized binders on certain properties of the mixtures also depends on the quality of the aggregates used. Some characteristics are not improved or even slightly worsens with rubberized asphalt mixtures containing reclaimed asphalt pavement^28^ or recycled concrete aggregates^29^, although it was proved that mixtures with volcanic aggregates and crumb rubber modified (CRM) bitumen offered superior water resistance and stability, and lower rut depth^30^. However, dynamic laboratory tests such as fatigue and stiffness tests have not been assessed yet for this type of rubberized warm mixtures with porous volcanic aggregates, although these properties are crucial for designing asphalt pavements.

Hence, to achieve more sustainable materials for pavement construction it is of vital importance to reduce the use of raw materials by replacing them with others that come from waste, and to decrease environmental impact by using available resources as well as reducing manufacturing temperatures at the asphalt mixing plant, which decreases fuel consumptions and emissions. Another goal is to increase pavement durability by improving the compaction and the mechanical performance of the mixtures. These are the reasons why this research aims to evaluate in the laboratory the performance of waste porous volcanic aggregates and scrap tire rubber on low-energy asphalt mixtures. This study is related to different dynamic properties and production characteristics of the asphalt mixtures manufactured at different low temperatures by using a surfactant liquid additive of easier dosage in industrial production of mixtures than granular wax additives.

## Materials and methods

### Aggregates

**Figure 1:**
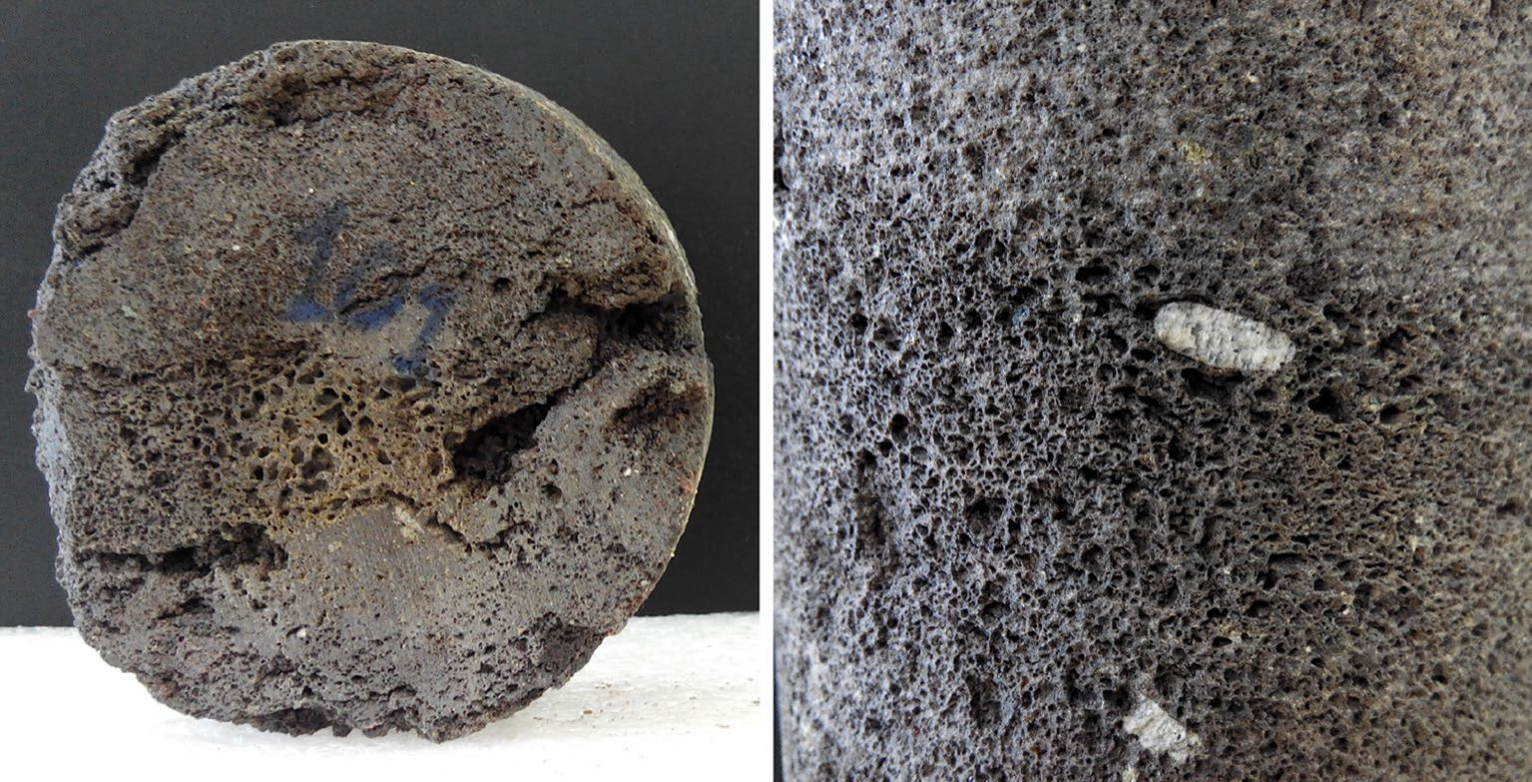
Cores from volcanic rock of the lithotype vesicular and scoriaceous basalt.

To guarantee consistency, the fractions of aggregate for the mixtures (0–4, 4–10 and 10–20 mm) were mechanically crushed from the same type of volcanic rock in the same quarry: an olivine-pyroxene grey basalt (B-V) of high porosity from the island of Gran Canaria (Spain). This type of aggregate is a very common and abundant lithotype of volcanic rock and considered to be as a marginal material, only used as a filling material but not suitable for structural concrete. Figure 1 shows the characteristic porous and scoriaceous structure of a rock sample. The geochemically classification of this rock is a basanite with brown amphibole, augite and phenocrysts of plagioclase and olivine.

**Figure 2:**
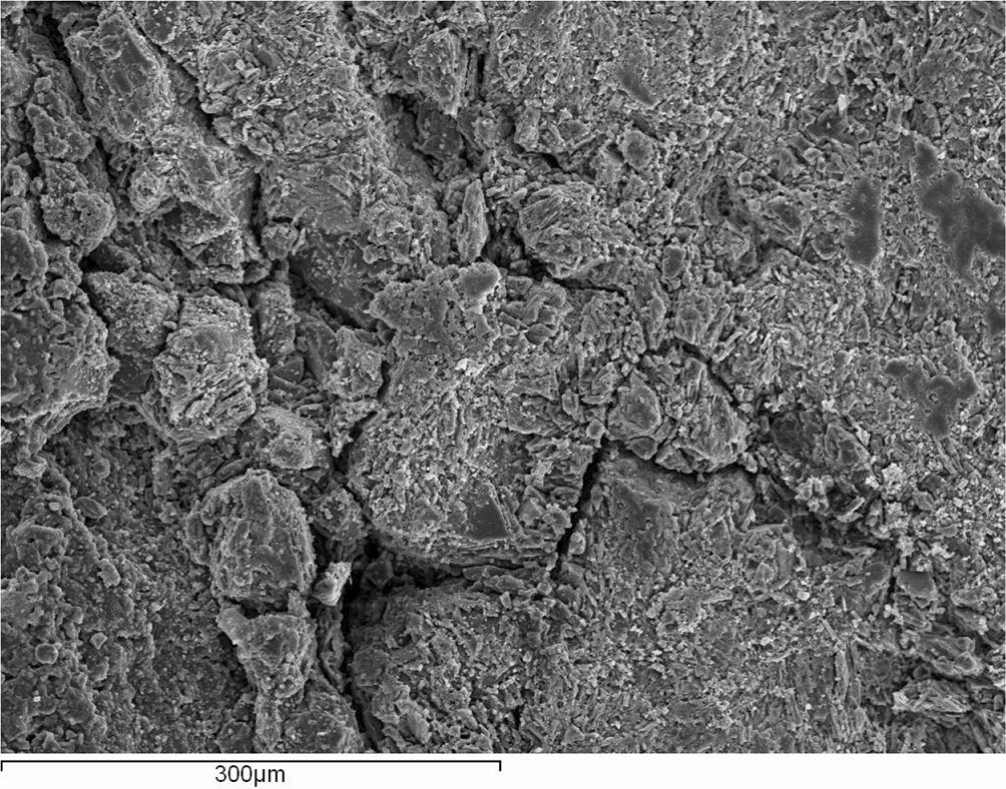
Mechanically crushed aggregate obtained from the vesicular basaltic rock: detailed image (magnification × 200) by electron microscope.

This aggregate offers high water absorption (WA_24_), especially in the finest fraction. Some properties complied with the Spanish specifications for road pavements: polished stone value (PSV), sand equivalent of the fraction 0–4 mm (SE_4_) and flakiness index (FI). Nevertheless, this volcanic aggregate is regarded to be marginal because of the high proportion of non-prismatic particles (particles with more than 50% of their surface crushed or broken, Cc = 56–60%), the resistance to fragmentation (Los-Angeles coefficient, LA = 28–29) and to wear (Micro-Deval coefficient, M_DE_ = 17–23). The finest fractions displayed lower particle densities and superior water absorption: WA_24_ of the fraction 0–4 mm was three times higher than the fraction 10–20 mm. More details of the main characterization properties of these marginal highly porous aggregates are shown in Table 1. Figure 2 shows a detailed electron microscope image of the aggregate microstructure. Moreover, as mineral filler (# < 0.063 mm), a Portland cement with pozzolanic addition (type CEM II/B-P 32.5 R according to EN 197-1) was used, since this cement is commonly produced in volcanic regions where natural pozzolans are plentiful.

**Table 1:** Physical and mechanical characteristics of the volcanic aggregate.

	Aggregate fraction			
	# 0–4 mm	# 4–10 mm	# 10–20 mm	Mineral filler (# < 0.063 mm)
Lithotype	Vesicular and scoriaceous grey basalt			100% CEM II/B-P 32.5 R [EN 197-1]
Proportion (% by wt. of total aggregate)	38.88	36.27	20.93	3.92
Physical properties				
Particle density [apparent]^1^	2450	2880	2890	–
Particle density [saturated surface dry]^2^	2360	2560	2630	–
Particle density [dry]^3^	2230	2370	2350	–
Water absorption of particles after 24 h^4^	15.5	8.3	5.8	–
Flakiness index^5^	–	6	6	–
Particles ≥ 50% of their surface crushed or broken^6^	–	60	56	–
Sand equivalent of finest fraction^7^	73	–	–	–
Mechanical properties				
Los-Angeles coefficient^8^	–	28	29	–
Micro-Deval coefficient^9^	–	23	17	–
Polished stone value^10^	–	60	60	–

^1^ρ_a_ (kg/m^3^) [EN 1097-6].

^2^ρ_SSD_ (kg/m^3^) [EN 1097-6].

^3^ρ_rd_ (kg/m^3^) [EN 1097-6].

^4^WA_24_ (%) [EN 1097-6].

^5^FI [EN 933-3].

^6^Cc [EN 933-5].

^7^SE_4_ [EN 933-8].

^8^LA [EN 1097-2].

^9^M_DE_ [EN 1097-1].

^10^PSV [EN 1097-8].

### Binders

The binder used for the conventional HMA reference mixture (control mixture without rubber) was a 35/50 penetration grade bitumen, whilst the binder for the other reference mixture (with rubber), a rubberized asphalt mixture named here as R-HMA, was a crumb-rubber modified binder of the same penetration grade, named here as CRMB 35/50. The CRM from used tires and the base bitumen (a 50/70 penetration grade bitumen) were blended with a proportion by weight of 10% CRM and 90% bitumen 50/70. The rubberized asphalt mixtures with the warm mix asphalt (WMA) additive are named here as R-WMA and the binder is the same: CRMB 35/50 with 0.5% (by the weight of the binder) of a chemical additive employed to reduce the production and compaction temperatures, commercially named Cecabase RT®. This WMA additive is a surfactant liquid product (water-free) and is composed of renewable components (at least 50%).

The CRM was previously manufactured using a mechanical grinder at room temperature (50% ELT from cars; 50% ELT from trucks). Only one batch of CRM was used to guarantee consistency. The composition of CRM (thermogravimetric analysis) is 57.41% of polymeric rubber and 32.22% carbon black; 6.02% are ash and 4.67% plasticizer and additives. 94% by weight of CRM had a size inferior to 0.5 mm.

To manufacture the CRMB 35/50, each 50/70 bitumen sample of 600 g was heated to 180 ± 1 °C. Afterwards 10% (by weight) of CRM was included in the blending unit with an oil bath and mixed for 60 min at 4000 rpm and 180 °C. In this way, the ultraviolet inhibitors, anti-oxidants and other chemicals present in the rubber shift to the asphalt, together with the elastomeric properties. The result was a reacted rubberized binder of higher consistency. To manufacture the binder for the R-WMA mixtures, then 0.5% of Cecabase RT® (by the weight of the CRMB 35/50) was carefully added to the rubberized bitumen and blended for 10 min at 4000 rpm and 180 °C, ensuring that the additive was properly incorporated into the binder. Bitumen density, penetration and softening point were tested according to EN 15326, EN 1426, and EN 1427, respectively. Dynamic viscosity was tested with a Brookfield rotational viscometer according to EN 13302. The main properties of the binders in the three types of asphalt mixtures are summarized in Table 2.

**Table 2:** Main properties of the asphalt bitumen for the different types of mixtures.

Type of asphalt mixture		HMA	R-HMA	R-WMA
Binder type	Penetration grade^5^	35/50	CRMB 35/50	CRMB 35/50
WMA additive		–	–	Cecabase RT®
Density^1^		1042	1028	1027
Penetration^2^		44	38	30
Softening point^3^		51.6	64.2	67.4
Viscosity^4^	At 60 °C	51,000	215,000	211,000
	At 135 °C	600	2100	2000
	At 150 °C	250	900	850

^1^*D* (kg/m^3^) [EN 15326].

^2^Pen at 25 °C, 100 g, 5 s. (× 10^−1^ mm) [EN 1426].

^3^Soft point, ring and ball test (°C) [EN 1427].

^4^Dynamic viscosity, Brookfield viscometer (cP) [EN 13302].

^5^Equivalence, for guidance purposes only, with the Performance Grade (PG): 35/50: PG 70-16; CRMB 35/50: PG 76-16 (note: however, that the properties required for PG classification have not been directly tested).

### Asphalt mixtures

In this study 174 cylindrical specimens, 60 prismatic specimens and 24 non-compacted samples (the latter for theoretical maximum density tests) were manufactured in the laboratory. As mentioned before, there are two reference mixtures: the conventional hot mixture (HMA) and the hot mixture with rubberized bitumen (R-HMA). A gradation of semi-dense asphalt concrete (AC16 surf S) was selected for all the mixtures tested in this research. This is a bituminous mixture used extensively for wearing course of different roads, types of traffic and climatic conditions because it provides a better surface macrotexture, lower deformation (due to its mineral skeleton and void content) and is cheaper to produce compared to dense asphalt concrete. The mixtures were manufactured according to the Spanish technical specifications for roads, PG-3^31^ and the European Standard EN 13108-1. In order to compare the effect of the CRM binders as well as the temperature reduction additive on the properties of this semi-dense asphalt concrete with highly-vesiculated basalt, three types of mixtures were produced:

- First, the reference HMA specimens were manufactured and tested. A 6% of bitumen content (by total weigh of mixture) was in the optimum range according to previous results of density, air voids, stability, flow value and resistance to plastic deformations of the mixtures (Marshall, ITSR, and wheel tracking tests).
- Secondly, the R-HMA specimens were produced and tested. These were produced with the identical type of aggregate, particle size distribution (see Fig. S1 in Supplementary Information) and content of binder (6%), but with a CRM binder of similar penetration grade, previously produced in the laboratory by blending the crumb rubber and a base bitumen 50/70 pen (penetration at 25 °C, 100 g, 5 s: 58 × 10^–1^ mm; softening point: 48.6 °C). With this last bitumen it was possible to obtain the CRMB 35/50, because the elastomer increased the viscosity and consistency of the resulting binder (reduced penetration by 34.5% and increased softening point by 32.1%), as previously stated by other researchers^32^.
- Thirdly, the warm mixtures with rubberized bitumen (R-WMA) and the same composition (6% of bitumen by wt. of mixture, including in this percentage the surfactant additive) were manufactured at different lower temperatures and tested. Manufacturing temperatures were between 140 and 170 °C, and compaction temperatures between 130 and 160 °C.

**Figure 3:**
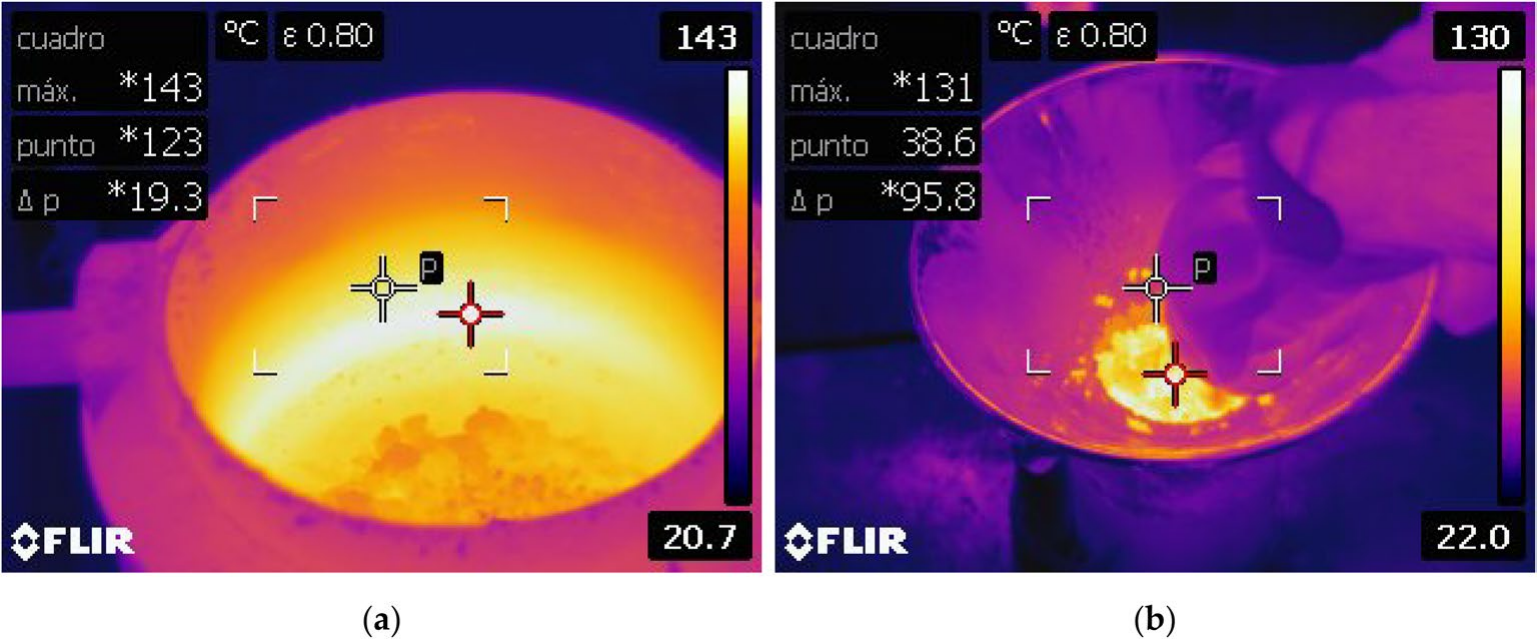
Thermal infrared imaging of the cylindrical specimen production of the R-WMA mixtures: (**a**) mixed at 140 °C; (**b**) compacted at 130 °C.

The reference HMA mixture was manufactured at 170 °C and the compaction temperature was 160 °C. The reference R-HMA mixture, due to the higher viscosity of the rubberized binder, was manufactured at 180 °C and the compaction temperature was 170 °C. Finally, the R-WMA mixtures were mixed between 140 and 170 °C and compacted between 130 and 160 °C. Compaction was always carried out at 10 °C below the corresponding mixture temperature (Fig. 3 presents thermal infrared imaging of the cylindrical specimen manufacturing of the R-WMA mixtures). Both the aggregates and the rubberized binder with the additive formerly incorporated were previously heated at the same temperature equal to the required mixing temperature and later coated with the bitumen for one minute by hand followed by 2 min in the mixer.

The cylindrical specimens of diameter 101.6 mm and height 63.5 mm were compacted by a Marshall hammer (EN 12697-30) with 50, 75 or 100 blows per side depending on the laboratory test (see details of the cylindrical specimens in Fig. S2 in Supplementary Information). The prismatic specimens for fatigue tests were produced from slab specimens of 300 × 300 × 60 mm, compacted by rolling in accordance with EN 12697-33. The compacted specimens and non-compacted samples received up to three series of tests for each mixture type and production temperature, with a minimum between three and six specimens for each test. Summarizing, a total of 258 laboratory specimens and samples were tested. The total number of experiments was: 76 characterization tests and 480 ultrasound tests. When required, the test samples were conditioned in a heater-refrigerator during the time to reach the normalized temperature according to standards and maintained during the test if necessary.

The different characterization tests are classified into the categories described below. For brevity, the equations used to calculate the properties of the different types of mixtures from the results of the dynamic characterization tests are included in the Supplementary Information.

- Volumetric properties: theoretical maximum density (according to EN 12697-5, Procedure A: volumetric); bulk density (according to EN 12697-6, Procedure B: saturated surface dry, and Procedure D: geometric); and void characteristics (according to EN 12697-8) on cylindrical specimens compacted by impact with different energies (number of blows) using the Marshall hammer, according to the Spanish specifications for asphalt concrete.
- Compaction properties: compactability test (according to EN 12697-10) on cylindrical specimens compacted by impact up to 2 × 100 blows (according to EN 12697-30) using the Marshall compactor, monitoring and recording the change of the specimen thickness during the compaction process (method of same sample for all energy levels).
- Dynamic mechanical performance: dynamic stiffness modulus (according to EN 12697-26, by indirect tensile test on cylindrical specimens [IT-CY] compacted by impact with 2 × 75 blows, k = 0.6, T = 20 °C, f = 2.2 Hz); elastic constants by determining the velocity of ultrasonic pulses (elasticity moduli and Poisson’s ratio at 20 °C, according to EN-12504-4 and BS 1881:Part 203); and resistance to fatigue (according to EN 12697-24, by four-point bending test on prismatic specimens [4 PB-PR] with 10^6^ cycles, at 20 °C, 10 Hz).

## Results and discussion

### Mixture manufacturing

#### Volumetric properties

Volumetric properties are normally the first characteristics studied in the design of a bituminous mixture because such characteristics are essential in its formulation and behaviour. Inadequate air void content and insufficient density may cause an impoverished mechanical performance^33^. If the air void content is excessive, it may cause accelerated water damage^34^ and reduced fatigue life^35^. On the contrary, if it is very low, heavy traffic can produce permanent deformations.

**Figure 4:**
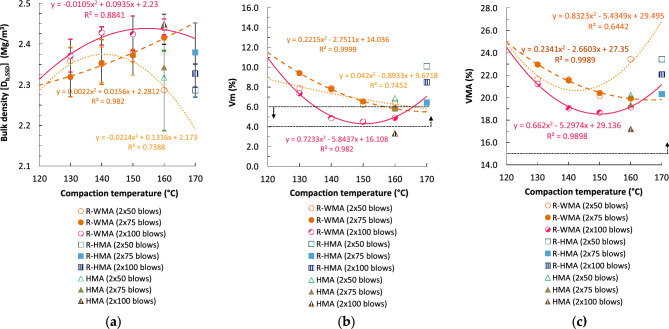
Volumetric properties with different compaction energies and temperatures on cylindrical specimens by Procedure B: saturated surface dry (SSD): (**a**) SSD bulk density; (**b**) air void content in the mixture; (**c**) voids in mineral aggregate.

**Figure 5:**
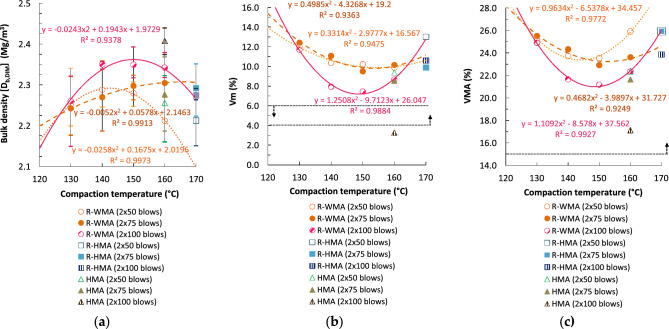
Volumetric properties with different compaction energies and temperatures on cylindrical specimens by Procedure D: geometric bulk density (DIM): (**a**) DIM bulk density; (**b**) air void content in the mixture; (**c**) voids in mineral aggregate.

Figures 4a and 5a present the bulk densities of cylindrical specimens compacted by impact with different energies (blows/side) versus the compaction temperature. In general, the bulk density of the R-WMA mixtures, determined by both the saturated surface dry [SSD] and the geometric [DIM] procedure, increased with the compaction energy (n° of blows), although in the case of the lower compaction temperatures (130 and 140 °C) no significant differences are observed for the densities obtained with the lowest energies (2 × 50 and 2 × 75 blows). This shows that the higher viscosity of the rubberized asphalt mixtures requires even higher compaction energies when they are manufactured and compacted at lower temperatures.

With the three compaction energies tested, the R-WMA mixtures allowed to achieve a density equal to or even higher (0.2–3.3% superior) compared to the reference R-HMA mixture, if the former are compacted at a temperature never inferior to 150 °C. However, in the case of using the highest compaction energy (2 × 100 blows), a higher density is attained even if the temperature is reduced to 140 °C. Therefore, the influence of the surfactant chemical additive on the compaction of the R-WMA mixtures is significant, allowing even higher densities than the reference R-HMA mixtures, even with a reduction of the compaction temperature of 20–30 °C. It should be noted that the densities of the R-WMA mixtures do not always increase with the compaction temperature; they were generally maximum at temperatures between 140 and 160 °C.

Compared to the reference HMA mixture, the densities of the R-WMA mixtures were similar or slightly higher when they were compacted at temperatures ≥ 140 °C, in the case of the lowest compaction energies (2 × 50 and 2 × 75 blows). On the contrary, when using the highest compaction energy (2 × 100 blows), all the R-WMA mixtures presented lower densities than the HMA reference mixture (between 2.47 and 6.55% lower, depending on the compaction temperature).

Table 3 outlines the averages of the statistical deviation regarding the results of the different properties. Bulk densities attained for both types of mixtures proved to be homogeneous, particularly in case of rubberized mixtures (coefficient of variation: Cv ≤ 1.94% for R-WMA mixtures; Cv ≤ 1.7% for R-HMA; and Cv ≤ 3.0% for HMA).

**Table 3:** Averaged statistical deviation parameters for the different properties tested.

Property of the mixture	HMA		R-HMA		R-WMA	
	Sd	Cv (%)	Sd	Cv (%)	Sd	Cv (%)
D_m,V_	100	3.98	30	0.99	20	0.62
D_b,SSD_ (2 × 50 blows)	40	1.62	20	0.89	40	1.53
D_b,DIM_ (2 × 50 blows)	40	1.93	40	1.57	40	1.60
D_b,SSD_ (2 × 75 blows)	60	2.74	40	1.71	50	1.94
D_b,DIM_ (2 × 75 blows)	70	3.03	30	1.45	50	1.89
D_b,DIM_ (2 × 100 blows)	30	1.18	20	1.06	40	1.80
T	10	14.15	13.08	32.51	6.67	20.11
S_m[IT-CY]_	375.34	5.98	521.69	8.48	622.8	7.84

*D*_*m,V*_ theoretical maximum density (kg/m^3^). *D*_*b,SSD*_ bulk density [SSD] (kg/m^3^). *D*_*b,DIM*_ bulk density [geometric] (kg/m^3^). *T* resistance to compaction by impact (blows). *S*_*m[IT-CY]*_ stiffness modulus by indirect tensile tests on cylindrical specimens (MPa). *Sd* standard deviation. *Cv* coefficient of variation.

The air void content (V_m_) of the mixtures was calculated comparing the theoretical maximum density and the bulk density. The theoretical maximum density depends on the aggregate type and gradation as well as on the bitumen type and content. These theoretical maximum densities of the rubberized mixtures were effectively higher compared to the reference mixture without rubber, for all the production temperatures studied (2.0–3.1% superior), although all mixtures were made with the same binder content (6% by total wt. of mixture).

Figure 4b,c present the void characteristics obtained by SSD bulk densities, and Fig. 5b,c the void characteristics with different compaction energies by geometric bulk densities. The air void content (V_m_) of the R-WMA mixtures presented a minimum value for compaction temperatures between 150 and 160 °C, depending on the method of determination of bulk density (SSD or DIM) and on the compaction energy (n° of blows), since the densities also presented maximum values in that temperature range. Voids in mineral aggregate (VMA) of R-WMA mixtures provided minimum values with temperatures between 140 and 160 °C, which means that the content of voids filled with binder (VFB) presented maximum values in the same range of compaction temperatures (for brevity, the VFB graphs are included in Supplementary Information, Fig. S3).

This is consistent with what was observed with the densities obtained: voids (both V_m_ and VMA) decreased with increasing compaction energy, although in the case of the lowest energies this only occurred with temperatures ≥ 150 °C. Voids in mixture (V_m_) were even inferior (between 9.6 and 25.1% lower) to those of the reference R-HMA mixture for most compaction energies, provided that the final temperature of compaction remains ≥ 150 °C; although for the maximum energy, the voids percentage of the R-WMA mixtures was always lower with any of the temperatures tested. Compared to the reference HMA mixture, the void content of the R-WMA mixtures was higher (between 8.9% and 166.4% superior) for practically all temperatures and compaction energies. The latter is due to the increased theoretical maximum density of the rubberized mixtures compared to the mixtures with conventional bitumen. Moreover, the void properties of R-WMA mixtures fulfilled the standard specifications for road pavements (4 ≤ V_m_ ≤ 6% and VMA ≥ 15%, in the case of asphalt concrete in wearing courses) for compaction temperatures ≥ 155 °C.

#### Compactability tests

**Figure 6:**
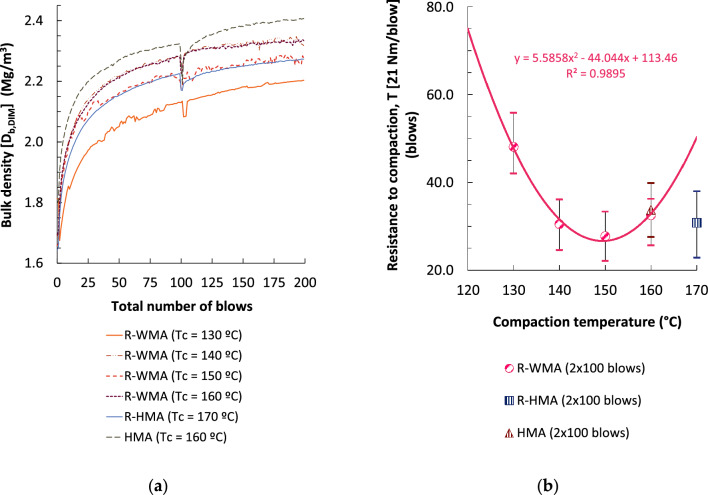
Compactability test on cylindrical specimens: (**a**) Geometric bulk density vs. number of blows; (**b**) resistance to compaction (specimens compacted with 100 blows per side).

Compactability results by the method of same sample for all energy levels (EN 12697-10) are shown in Fig. 6. As previously stated, the geometric bulk density of R-WMA cylindrical specimens compacted by the Marshall compactor with the maximum energy (100 blows per side) increased with the compaction temperatures. The geometric densities obtained with this maximum energy for the R-WMA mixtures were equal or greater than that of the reference R-HMA mixture, compacted at 170 °C, even when compaction temperature was as low as 140 °C. Compared to the reference HMA mixture, they presented lower geometric density in the entire range of temperatures tested.

The same Fig. 6 also shows the resistance to compaction by impact (T) of cylindrical specimens up to 2 × 100 blows. This T value of the R-WMA mixtures was minimum at 150 °C, which is consistent with the compaction temperature that also provided minimum void content in the mixture and maximum geometric bulk density, being T even inferior (9.9% lower) to that obtained with the reference R-HMA mixture (compacted at 170 °C). When compared to the conventional HMA mixture, the R-WMA mixtures showed a lower T value up to temperatures as low as 140 °C, which also shows the favourable effect of the surfactant additive.

### Stiffness modulus

**Figure 7:**
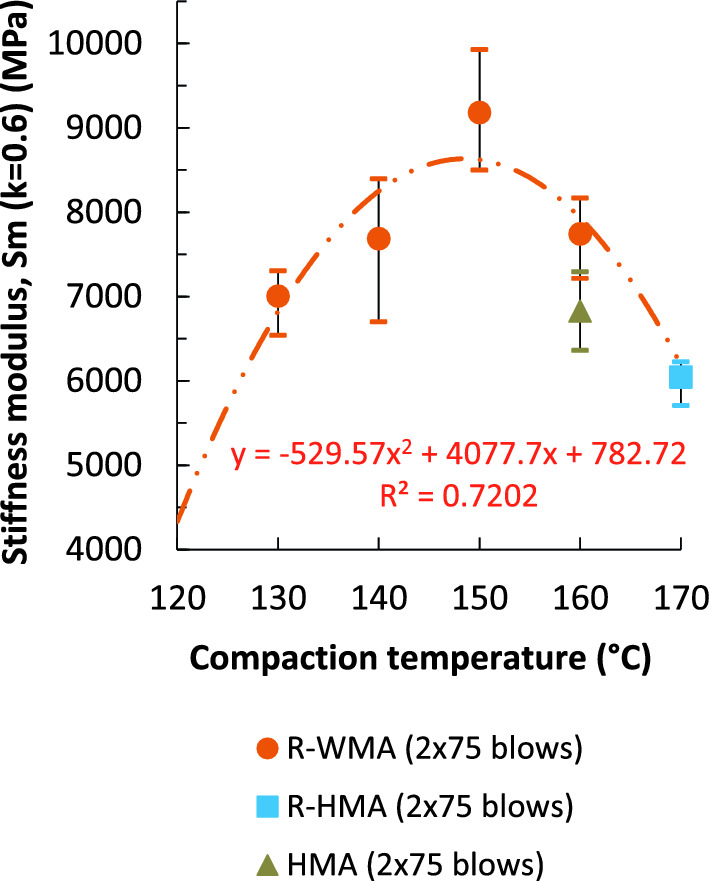
Dynamic stiffness modulus by indirect tensile test on cylindrical specimens.

The stiffness of a bituminous mixture varies with multiple factors: the type of mixture, aggregate and bitumen, the mixture gradation, the bitumen content, the mixture density and the air void content, among others. Dynamic stiffness modulus (S_m_) by indirect tensile tests on cylindrical specimens (IT-CY, load surface factor k = 0.6, at 20 °C, f = 2.2 Hz; compacted with 75 blows per side) are shown in Fig. 7. According to laboratory results, the stiffness modulus of the R-WMA mixtures showed a maximum value when compacted at 150 °C. This modulus was superior to both reference mixtures, for all the compaction temperature range that was studied. At 150 °C the result was 51.9% and 34.5% superior to R-HMA and to HMA, respectively. But even when compacted at a temperature as low as 130 °C, this modulus was 15.9% and 2.6% higher, respectively. The laboratory results present a statistical dispersion with an average coefficient of variation (Cv) below 8.5% in any case, as it can be seen in Table 1, thus showing sufficient homogeneity.

In the entire range of compaction temperatures, the stiffness moduli of the R-WMA specimens resulted superior to 7000 MPa. A reference value commonly considered in the analytical design of pavement structures for asphalt concrete AC16 surf S mixtures is usually around 6000 MPa. Consequently, all mixtures studied in this research would meet the stiffness requirements for use in the construction of road pavements, even those manufactured at the lowest temperatures. These results also show that the increase in stiffness of the R-WMA mixtures, that can be observed at any compaction temperature, is not due to the use of the rubberized binder, as other authors concluded^30^, since the reference R-HMA mixture with the same rubberized bitumen provided even a lower stiffness modulus compared to the reference HMA mixture with conventional bitumen. The experimental results demonstrate that the mentioned increase in stiffness, even at low temperatures, may be due to the action of the chemical surfactant additive, since in this study the stiffness modulus correlates very closely with the volumetric properties of the mixtures.

### Fatigue performance

**Figure 8:**
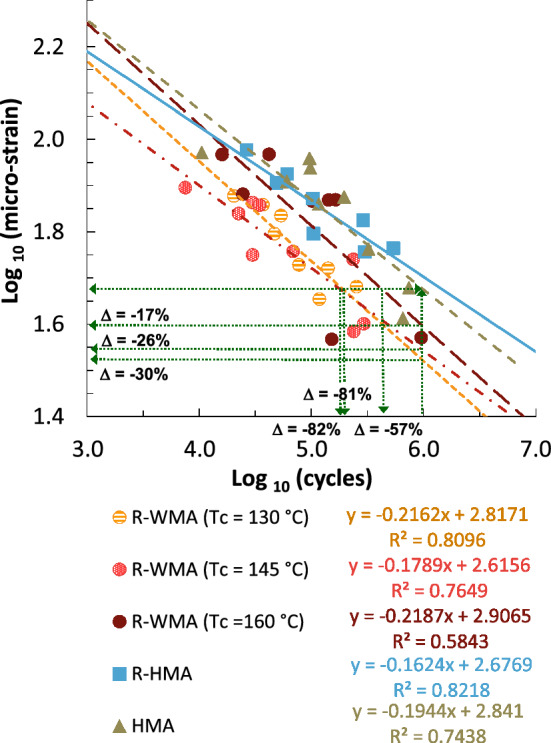
Fatigue laws by four-point bending test on prismatic specimens.

Cracking resistance of asphalt mixtures depends on their ductility, extensibility, and tension strength. These properties vary mainly with the type and content of asphalt binder and with the mixture stiffness. Fatigue laws under repeated loads by four-point bending test on prismatic specimens (4 PB-PR, at 20 °C, 10^6^ cycles, 10 Hz) of the different mixtures are summarized in Fig. 8. In order to reduce the large quantity of specimens required for these tests, the two intermediate compaction temperatures under this study (140 °C and 150 °C) were simplified to 145 °C, maintaining both extreme temperatures (130 °C and 160 °C), resulting in 60 prismatic specimens.

As can be observed, the resistance to fatigue of the R-WMA specimens is reduced as the mixtures were compacted at lower temperatures with the WMA additive. The initial micro-strain for an expected service life until failure of 1 × 10^6^ cycles of the R-WMA mixtures decreased in regard both reference mixtures (R-HMA and HMA). Compared to the HMA mixture it decreased by 17, 26 and 30%, when the R-WMA mixtures were compacted at 160, 145 and 130 °C, respectively. This represents a decrement of the expected number of fatigue cycles of 57, 81 and 82%, also respectively, to reach the aforementioned micro-strain. Despite this reduction in fatigue resistance of the R-WMA mixtures when produced at lower temperatures, images from electron microscope have shown that fatigue micro-cracks present fine microscopic ridges and well pronounced dimples, thus with no smooth fracture surfaces and consequently, avoiding brittle fracture (see Fig. S4 in Supplementary Information).

A comparative analysis of the fatigue laws of these asphalt mixtures with respect to mixtures with conventional aggregate suggests a decrease in the number of load cycles until fatigue failure, which can be estimated between 67 and 89% on average. However, in extremely sensitive regions such as the volcanic islands, it is crucial to advance in the sustainability of asphalt pavements by reducing the use of raw materials and recycling waste materials. In this case, the inferior fatigue performance due to the material properties must be compensated at the design stage by modifying other pavement parameters such as layer thickness and the arrangement of the different materials in the different layers.

### Elastic constants determined by ultrasounds

The feasibility of ultrasonic methods in asphalt mixtures has been sparsely used, due to the characteristic heterogeneity and air void content of these materials. Some studies used direct transmission methods measuring ultrasonic pulse velocity of P-waves (compression waves) and S-waves (shear waves) to obtain the elastic constants of cylindrical specimens^36^. In our research we have attempted to verify the feasibility of determining these elastic constants by means of ultrasounds in the case of asphalt mixtures with an extremely porous mineral aggregate such as the one used in this study. Subsequently, the elastic moduli obtained by ultrasonic measurements have been correlated with those previously obtained in the dynamic tests of stiffness moduli.

**Figure 9:**
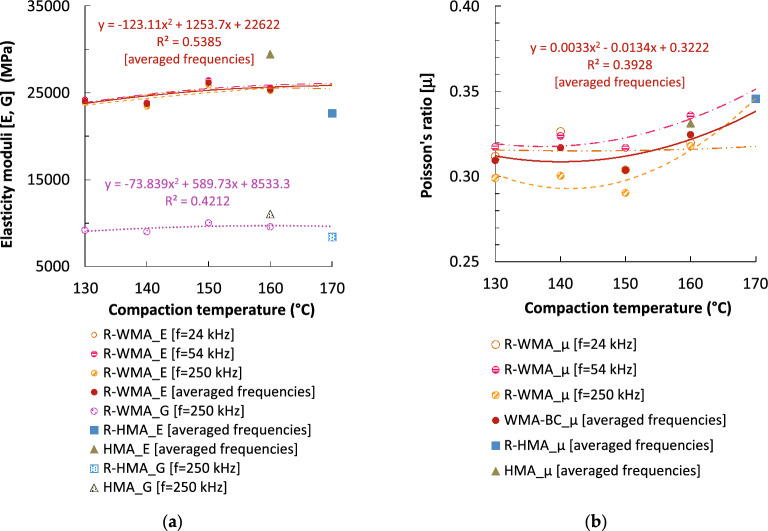
Ultrasound elastic constants (measured at 20 °C and different ultrasonic frequencies on impact-compacted cylindrical specimens with 75 blows/side): (**a**) Young’s modulus (E) and shear modulus (G); (**b**) Poisson’s ratio (μ).

The elastic constants were determined at a room temperature of 20 °C (the same as the stiffness tests) from the results measured of ultrasound velocities on the same cylindrical specimens used for stiffness tests, and employing transducers of three different frequencies. Figure 9 summarizes the elastic constants of the R-WMA mixtures compacted at different temperatures and compares them with both reference mixtures. Minor differences were observed varying the ultrasonic pulse frequency, all of them limited frequencies (24, 54 and 250 kHz). Both the Young's modulus (E) and the Shear modulus (G) showed a similar trend with the variation of the compaction temperature to that observed with the dynamic stiffness modulus (Fig. 7), where the maximum value was obtained at 150 °C. The latter suggests that there could be a certain correlation between the elastic constants determined by ultrasound and the stiffness moduli obtained by dynamic stiffness tests.

**Figure 10:**
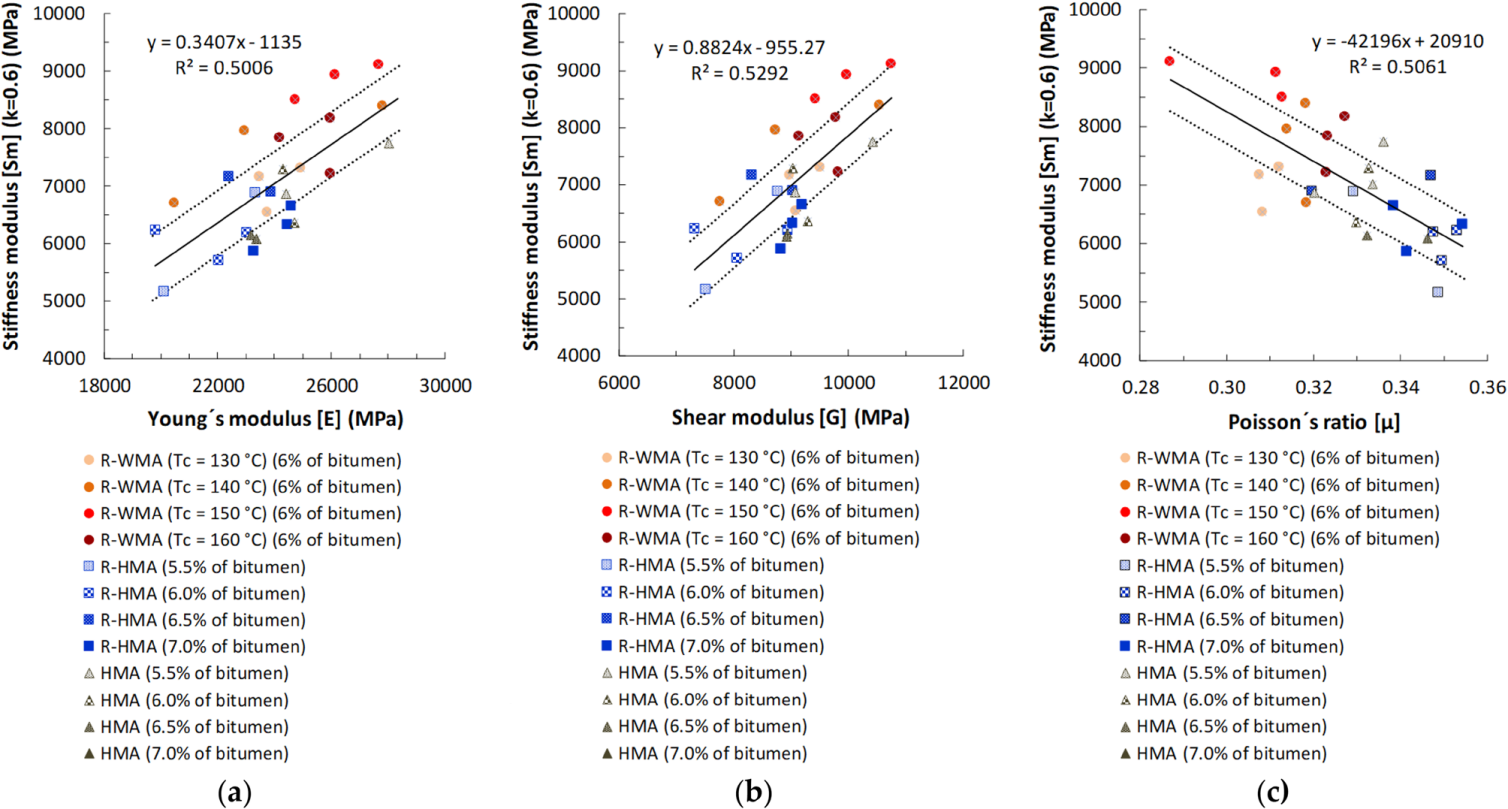
Correlations between the dynamic stiffness modulus by indirect tensile test and the elastic constants obtained by ultrasounds at 20 °C on the same cylindrical specimens. Averaged results for all pulse frequencies: (**a**) Young’s modulus (E); (**b**) Shear modulus (G); (**c**) Poisson’s ratio (μ) [Dotted lines represent the Standard Error limits of the linear fitting].

The correspondences obtained between the stiffness modulus by indirect tensile tests (S_m_) and the three elastic constants determined by ultrasounds (E, G and μ) are represented in Fig. 10. The experimental results show the existence of certain correlations between these magnitudes, although the coefficients of determination (R^2^) of the fitting are not high enough to infer that ultrasonic testing constitutes, in practice, an alternative or substitutive method to dynamic stiffness tests. This is due to the high statistical dispersion of ultrasound measurements in these highly heterogeneous and porous materials. However, the clear trend shown by the correlations, somewhat higher in the case of the shear modulus (G), suggests that the determination of the elastic constants of these asphalt mixtures, by measuring the propagation velocities of ultrasonic waves, could be used as an additional procedure for estimating the stiffness moduli of these materials. Without replacing dynamic stiffness tests, ultrasonic tests could complement the former, since they allow many more results to be obtained, faster, easier and with a reduced cost. By adapting the measurement method, they could even be used for non-destructive in situ determination on in-service pavements if calibrated models are previously obtained.

## Conclusions

The extraction of natural resources is becoming increasingly limited and it is therefore vital to make efficient and environmentally friendly use of available raw materials to meet the increasing demand in construction. According to this experimental study, the main conclusions are as follow:

- The combination of end-of-life tire rubber powder to modify the asphalt binder and a warm mix asphalt (WMA) additive makes it possible to reuse residual porous volcanic aggregates, while reducing production temperatures, complying with technical specifications for paving materials, and offering a promising solution to produce more sustainable materials.
- The rubberized warm asphalt mixtures (R-WMA) require higher compaction energy when manufactured and compacted at lower temperatures.
- The R-WMA mixtures achieved a density equal or even superior to the same hot mixture (R-HMA, compacted at standard temperature of 170 °C), if they are compacted never below 140–150 °C. Therefore, the influence of the surfactant chemical additive is significant, allowing higher densities even with a reduction of the final temperature of 20–30 °C.
- The air void content of the R-WMA mixtures was even inferior to the reference R-HMA mixture for all compaction energies if the final temperature of compaction does not fall below 150 °C. However, in case of maximum impact energy, the void percentage was always lower at any temperature and therefore, producing sufficiently dense mixtures.
- The resistance to compaction by impact of the R-WMA mixtures was inferior to that obtained with the R-HMA mixture (compacted at 170 °C). When compared to the conventional HMA mixture, they showed lower resistance to compaction even at temperatures as low as 140 °C, thus making easier the placement of the pavement.
- The stiffness modulus of the R-WMA mixtures was superior to both reference mixtures (35–52% superior at 150 °C). But even when compacted at a temperature as low as 130 °C, this modulus was between 3 and 16% higher. All the mixtures studied in this research would meet the required stiffness for pavement construction, even those manufactured at the lowest temperatures.
- Despite the observed reduction in fatigue resistance of these R-WMA mixtures when produced at lower temperatures, fatigue micro-cracks presented microscopic ridges and well pronounced dimples, thus with no smooth fracture surfaces and consequently, avoiding brittle fracture.
- Correlations between the stiffness modulus by indirect tensile test and the elastic constants determined by low-frequency ultrasound were obtained. They could be used in practice as a complementary procedure in order to pre-estimate moduli in an easier, faster and more affordable way.
- Finally, it can be stated that, even with these poor-quality aggregates, the dynamic properties of these R-WMA mixtures are maintained or even improved with reductions of the manufacturing and compaction temperatures of 20 °C, therefore diminishing emissions around 20%, saving fuel consumption nearby 25%, and saving virgin raw materials (binder and aggregates) more than 95% by weight.

## Supplementary Information

Supplementary Information.

## Data Availability

All data generated or analysed during the laboratory tests are included in this published article (and its Supplementary Information files). Raw data registered directly by testing instruments are available from the corresponding author under request.
