# Supplementary material for: Cleaner technologies for asphalt mixtures combining reuse of residual aggregates, waste crumb rubber and warm mix asphalt additive
Source: Sci Rep. 2023 May 19;13:8153. doi: 10.1038/s41598-023-35235-z (PMC10198975; doi:10.1038/s41598-023-35235-z)
Supplement: Supplementary file 1 — Supplementary Information. [file 41598_2023_35235_MOESM1_ESM.pdf]

Cleaner technologies for asphalt mixtures combining reuse of residual aggregates, waste crumb rubber and warm mix asphalt additive

Miguel A. Franesqui [a]*, Ana María Rodríguez-Alloza [b], Jorge Yepes [c], Cándida García-González [a]

[a] Grupo de Fabricación Integrada y Avanzada – Departamento de Ingeniería Civil, Universidad de Las Palmas de Gran Canaria (ULPGC), Campus de Tafira, 35017 Las Palmas de Gran Canaria, Spain.

[b] Grupo de Tecnología de Materiales en la Arquitectura y la Construcción (TermaCon) – Departamento de Ingeniería Civil, Náutica y Marítima, Universidad de La Laguna (ULL), Av. Ángel Guimerá Jorge, 1, 38206 San Cristóbal de La Laguna, Spain.

[c] Departamento de Ingeniería Civil – IOCAG, Universidad de Las Palmas de Gran Canaria (ULPGC), Campus de Tafira, 35017 Las Palmas de Gran Canaria, Spain.

* Corresponding author: miguel.franesqui@ulpgc.es (M.A. Franesqui).

Supplementary Information

1. Figures

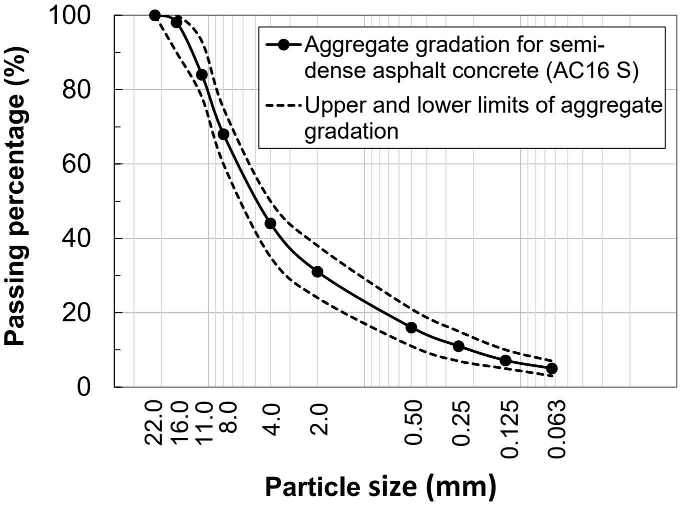

Supplementary Figure S1. Particle size distribution curve and specified grading envelope (semi-dense asphalt concrete of maximum aggregate size 16 mm).

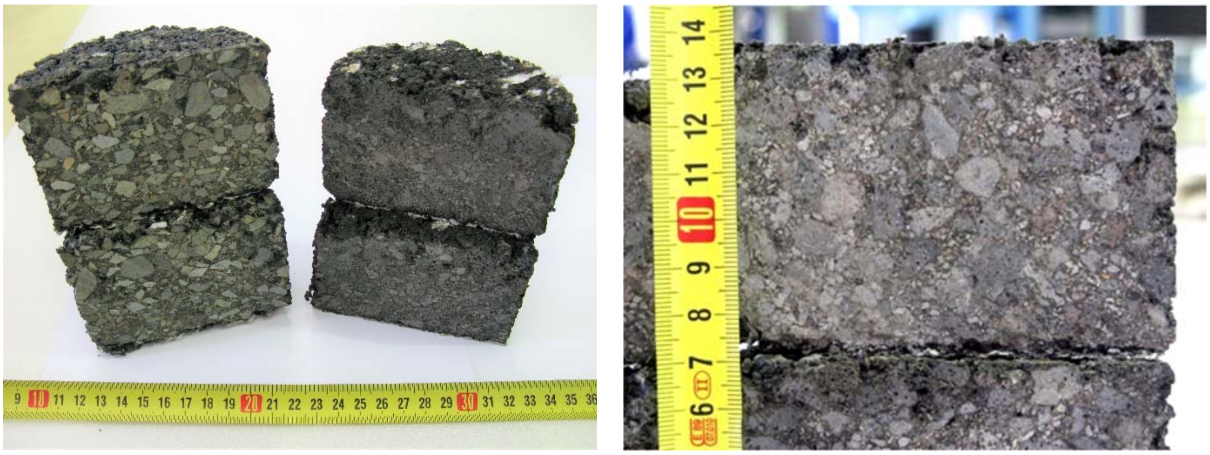

a) b)

Supplementary Figure S2. Details of cut section of cylindrical specimens of the three types of mixtures with vesicular volcanic aggregate tested in the laboratory: a) left: HMA mixture; right: R-HMA mixture; b) R-WMA mixture compacted at 140 ºC.

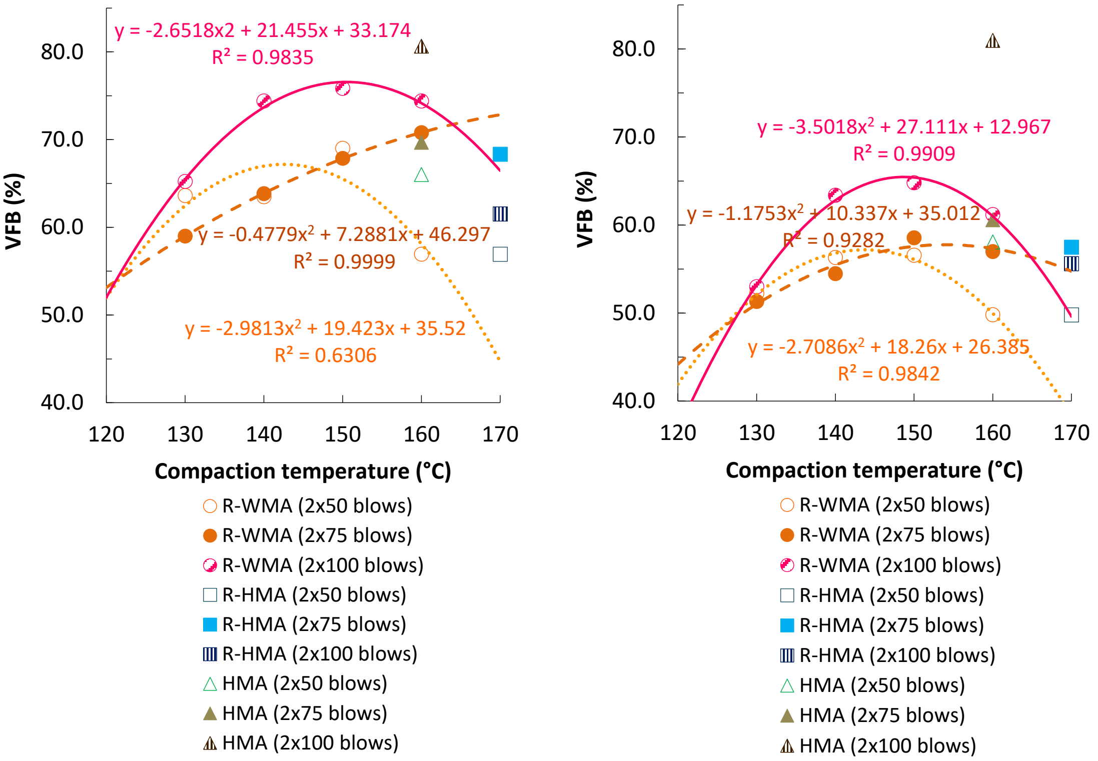

a) b)

Supplementary Figure S3. Voids filled with bitumen (VFB) with different compaction energies and temperatures on cylindrical specimens: a) Calculated from SSD bulk densities; b) Calculated from geometric (DIM) bulk densities.

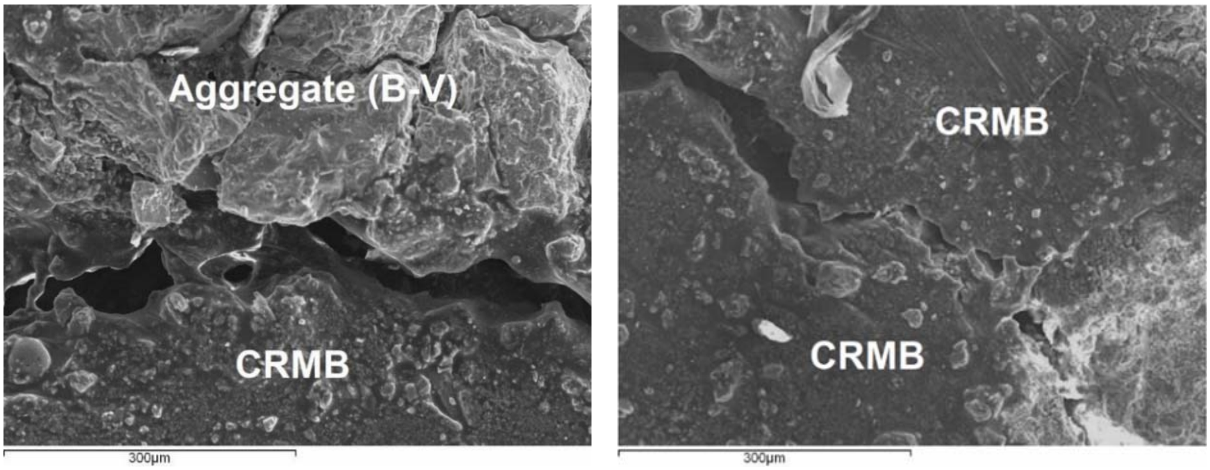

a) **b)**

Supplementary Figure S4. Images from scanning electron microscopy [magnification x200]: a) Fatigue micro-crack at the interfacial contact between the volcanic aggregate particle of type vesicular basalt and the crumb-rubber modified binder (CRMB); b) Fatigue micro-crack in the CRMB.

2. Equations for dynamic properties from characterization tests

2.1. Compactability test

Standard: EN 12697-10

Procedure:

Method of same sample for all energy levels on cylindrical specimens compacted by impact up to 2 x 100 blows (100 blows per each side) using the Marshall compactor (EN 12697-30), monitoring and recording the change of the specimen thickness during the compaction process.

Equation (1) relates the thickness variation of the specimen, the compaction energy applied and the resistance to compaction:

$$\frac{1}{t(E)} = \frac{1}{t_\infty} - \left(\frac{1}{t_\infty} - \frac{1}{t_0} \right) \cdot \exp\left(\frac{-E}{T} \right) \qquad (1)$$

where: t(E) = thickness of the compacted specimen as a function of the compaction energy (mm); t_∞ = minimum possible thickness of the specimen (mm); t_0 = initial thickness of the specimen (mm); E = compaction energy by impact, expressed considering 21 Nm as the unit (number of blows); T = resistance to compaction by impact (number of blows).

2.2. Stiffness modulus test

Standard: EN 12697-26

Procedure:

By indirect tensile test on cylindrical specimens [IT-CY] compacted by impact with 2 x 75 blows, k = 0.6, T = 20 ºC, f = 2.2 Hz) and the stiffness modulus obtained according to the equation (2).

In each IT-CY test five haversine repeated loading pulses with the corresponding intermediate rest periods were applied, controlling the loading time during tests. The ratio of rest periods to loading time (R/D) were between 20.5 and 22.2 (R/D should be equal or greater to 9 to achieve an acceptable range of error in measurement of resilient modulus). The load surface factor (k, related to the shape of the loading pulse curve or waveform), rise time (from zero load up to peak load), strain and stiffness modulus in each loading pulse were also obtained.

$$S_m = \frac{F \cdot (\mu + 0.27)}{(z \cdot h)} \tag{2}$$

where: S_m = Stiffness modulus measured by the test (MPa); F = maximum vertical load applied (N); z = horizontal strain amplitude during the loading cycle (mm); h = average specimen thickness (mm); μ = Poisson ratio (a constant value of 0.35 was assumed). According to EN 12697-26, it was verified that the rise time was between 120 and 128 ms, the load surface factor (k) between 0.5 and 0.8, and the strain between 3 and 20 μm. The stiffness modulus measured was corrected by Equation (3) when k ≠ 0.6.

$$S'_m = S_m \cdot \left\{1 - 0.322 \cdot \left[Log\left(S_m\right) - 1.82\right] \cdot \left[0.6 - k\right]\right\} \tag{3}$$

where: S'_m = Stiffness modulus corrected for a load surface factor of 0.6 (MPa); k = load surface factor measured.

Moreover, as this is a non-destructive test, each specimen was tested at two different diametrically opposite positions. The load amplitude was 2700 N, the pulse frequency 2.2 Hz and the resting time 2750 ms and the temperature during the test was 20 ºC. Afterwards, averages of these moduli were calculated as well as the mean values for each set of the three specimens with the same bitumen content.

2.3. Fatigue resistance test

Standard: EN 12697-24

Procedure:

By four-point bending test on prismatic specimens [4PB-PR] with 10^6 cycles, at 20 ºC, 10 Hz.

In each 4PB-PR test a repeated haversine load was applied on prismatic specimens. The four supporting points enable a flexural load with a constant strain within the two intermediate clamps of the beam specimen. The prismatic beams, obtained by cutting slab specimens, were tested at 20 ºC. According to EN 12697-24, the initial stiffness modulus calculated for each mixture specimen (S_{mix}) is obtained from the load, displacement and phase angle after 100 load applications. The test with strain control continues until the modulus diminishes up to half its initial value or up to

specimen failure. The fatigue law for fatigue life prediction was obtained by linear regression of the logarithms of the number of cycles and the logarithms of the initial strain amplitude, according to:

$$Log\left(\varepsilon_0\right) = a_0 + a_1 \cdot Log\left(N\right) \tag{4}$$

where: ε_0 = initial strain amplitude; N = load cycles until fatigue failure; a_0, a_1 = parameters (material constants) obtained from the 4PB-PR fatigue test.

2.4. Ultrasound test

Standard: EN-12504-4 and BS 1881:Part 203

Procedure:

Elastic constants determined by the Ultrasonic Pulse Velocity (UPV) method.

The elastic constants of the asphalt mixtures (elasticity moduli and Poisson's ratio at 20 ºC) were assessed by measuring the velocity of ultrasonic pulses on the same cylindrical specimens before the stiffness modulus tests, since the ultrasonic test are non-destructive. These tests were performed using a direct transmission scheme, placing each pair of piezoelectric transducers at the middle point of the bases of each cylindrical specimen. Transducers of three different low frequencies were used (24, 54 and 250 kHz), in order to avoid scattering and dispersion of waves by the aggregate particles and pores. Velocities of ultrasonic P-waves (longitudinal compression waves) and S-waves (transverse shear waves) were registered at a constant temperature of 20 ºC (in order to compare results with the stiffness modulus tests), because moduli of bituminous materials are temperature-dependent. According to BS 1881: Part 203 and ASTM D2845-00, the ultrasonic elastic constants of the mixtures can be obtained as follows, assuming an elastic medium:

$$G = D_b \cdot V_S^2 \tag{5}$$

$$E = G \cdot \frac{(3 \cdot V_P^2 - 4 \cdot V_S^2)}{V_P^2 - V_S^2} \tag{6}$$

$$\mu = \frac{(V_P^2 - 2 \cdot V_S^2)}{2 \cdot \left(V_P^2 - V_S^2\right)} \tag{7}$$

where: D_b = bulk density of the material (kg/m^3); V_S = shear-wave velocity (m/s); V_P = compression-wave velocity (m/s); G = shear modulus (Pa); E = Young´s modulus (Pa); μ = Poisson´s ratio.
